# Pyrogallol B-ring enhances catechin binding to the SARS-CoV-2 spike receptor-binding domain to inhibit interaction with ACE2

**DOI:** 10.1038/s41598-026-41170-6

**Published:** 2026-02-28

**Authors:** Futaba Matsumoto, Satomi Nagai, Nanako Ikeda, Kanji Ishimaru, Kozue Sakao, Takeshi Miyata, Yoichiro Hama, Susumu Mitsutake

**Affiliations:** 1 Graduate School of Advanced Health Sciences, Saga University, Saga, Japan; 2 Department of Life Science and Food Science, Faculty of Agriculture, Saga University, Saga, Japan; 3 Applied Bioscience, Faculty of Agriculture, Saga University, Saga, Japan; 4 The United Graduate School of Agricultural Sciences, Kagoshima University, Kagoshima, Japan; 5 Department of Applied Biochemistry and Food Science, Faculty of Agriculture, Saga University, 1 Honjo-machi, Saga, 840-8502 Japan

**Keywords:** Virology, Biochemistry, Viral infection

## Abstract

Severe acute respiratory syndrome coronavirus 2 infects host cells through binding of the spike protein receptor-binding domain (RBD) to the human angiotensin-converting enzyme 2 receptor. In this study, the antiviral activity of 14 catechin derivatives was evaluated using a pseudovirus assay that emulates spike-mediated cell fusion. Of these, gallocatechin gallate, epigallocatechin gallate, epigallocatechin 3-(3″-O-methyl) gallate, and epigallocatechin exhibit strong inhibitory effects on infection. A structural comparison of the compounds revealed that catechins with a pyrogallol-type B-ring configuration exhibited greater inhibitory effects than their catechol-type counterparts. Docking simulations demonstrated that the hydroxyl group at the 5-position of the B-ring forms a hydrogen bond with Gln493 on the spike RBD, thereby facilitating additional stabilizing interactions with adjacent residues, such as Tyr453. Although catechin bioavailability is low, the results of this study suggest that regular consumption or gargling may offer localized antiviral activity at mucosal surfaces, such as those found in the oral or nasal cavity, because the catechin concentrations used in the cell assays are similar to those observed in green tea (100 µM). This study underscores the potential of pyrogallol-type catechins to act as antiviral agents.

**Supplementary Information:**

The online version contains supplementary material available at 10.1038/s41598-026-41170-6.

## Introduction

In 2020, the World Health Organization declared the outbreak of severe acute respiratory syndrome coronavirus 2 (SARS-CoV-2) a global pandemic. The development of effective preventive and therapeutic strategies against SARS-CoV-2 remains a critical component of preparedness for current and future pandemics. SARS-CoV-2 enters host cells through two primary routes: (1) an endocytic pathway and (2) a non-endocytic mechanism involving direct membrane fusion at the cell surface, activated by the transmembrane serine protease 2 (TMPRSS2)^1,2^. Regardless of the entry route, the first step of infection is the binding of the spike protein receptor-binding domain (RBD) to the host angiotensin-converting enzyme 2 (ACE2) receptor to form a complex^3,4^. Therefore, inhibiting the RBD-ACE2 interaction represents a promising strategy to block viral entry and prevent infection. Epigallocatechin gallate (EGCG), the most abundant catechin in green tea, has demonstrated broad-spectrum antiviral activity in vitro, including the inhibition of multiple viruses such as influenza, human immunodeficiency, hepatitis B and C, chikungunya, vaccinia, herpes simplex, and vesicular stomatitis viruses, adenovirus, and reovirus^5–10^. EGCG has also shown potential in inhibiting SARS-CoV-2 infection^11–15^. This antiviral activity is attributed to EGCG’s ability to interact with various biomolecules, including proteins, lipids, and carbohydrates^16–18^. Accordingly, multiple mechanisms have been proposed to explain its antiviral activity. For instance, EGCG has been shown to bind to heparan sulfate on the surface of host cells and inhibit viral entry, as demonstrated using a lentiviral pseudoparticle system^12,19^. In addition, catechins have been reported to inhibit the activity of recombinant SARS-CoV-2 main protease (Mpro)^20^, suggesting a possible mechanism for suppressing viral replication. Furthermore, in silico analyses have indicated direct interactions between catechins and the SARS-CoV-2 spike protein RBD^21^. This has been experimentally supported by pull-down assays, which demonstrated that EGCG disrupts binding between the RBD and ACE2^22^. Considering that the RBD-ACE2 interaction represents a critical initial step for viral entry, we focused our investigation on this interaction to evaluate the inhibitory effects of various catechins on SARS-CoV-2 infection.

**Fig. 1:**
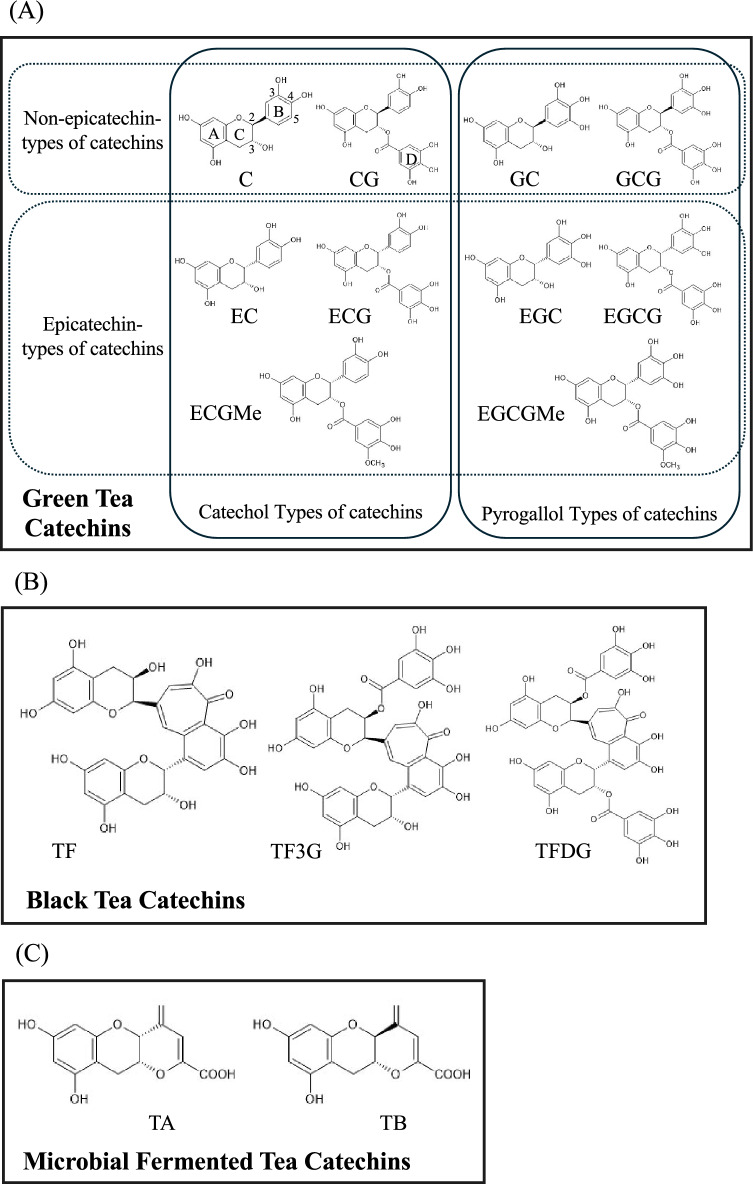
Catechins and the derivatives examined in this study. Catechins are composed of A- and B-ring benzene structures and a C-ring heterocycle, and are classified on the basis of C-ring stereochemistry, B-ring hydroxylation pattern, and the presence of a galloyl group at C3. (−)-Catechin (CH, CID: 107957), (−)-epicatechin (EC, CID: 72276), (−)-catechin gallate (CG, CID: 6419835), (−)-epicatechin gallate (ECG, CID: 107905), and (−)-epicatechin 3-(3″-O-methyl) gallate (ECGMe, CID: 467296) are classified as catechol-type catechins. (−)-Gallocatechin (GC, CID: 9882981), (−)-epigallocatechin (EGC, CID: 72277), (−)-gallocatechin gallate (GCG, CID: 199472), (−)-epigallocatechin gallate (EGCG, CID: 65064), and (−)-epigallocatechin 3-(3″-O-methyl) gallate (EGCGMe, CID: 467296) are categorized as pyrogallol-type catechins. Theaflavin (TF, CID: 134503798), theaflavin 3-gallate (TF3G, CID: 169167), and theaflavin 3,3′-di-O-gallate (TFDG, CID: 58252602) are generated through tea leaf fermentation. Teadenol A (TA, CID: 68196392) and teadenol B (TB, CID: 68196394) are obtained from tea produced by fermentation caused by microbes.

Tea is broadly categorized into non-fermented green tea, semi-fermented oolong tea, fermented black tea, and tea fermented by microbial processes, with each containing different catechins^23^. Catechins consist of two benzene rings (A-ring and B-ring) and a dihydropyran heterocycle (C-ring). They are classified according to three factors: (1) the stereochemical configuration at the C2 position of the C-ring, (2) the number and distribution of hydroxyl groups on the B-ring, and (3) the presence or absence of a galloyl group (D-ring) at the C3 position of the C-ring (Fig. 1). During tea processing, isomerization reactions occur at the C2 position of the C-ring, resulting in the existence of (−)-catechin (C) and (−)-epicatechin (EC)^24,25^. Catechins/epicatechins with hydroxyl groups at positions 3 and 4 of the B-ring are classified as catechol-type, whereas those with an additional hydroxyl group at position 5 are classified as pyrogallol-type. These include gallocatechin (GC) and epigallocatechin (EGC). In addition, esterification of (−)-catechin by gallic acid, a plant-derived organic compound, produces galloylated catechins such as ECG and EGCG. Methylated catechins, such as ECGMe and EGCGMe, are derivatives in which the 3-position of the galloyl group in ECG and EGCG is methylated, leading to improved chemical stability and membrane permeability. As a result, these compounds may exhibit enhanced biological activity compared with their non-methylated counterparts. Theaflavins are catechin dimers formed by enzymatic oxidative condensation between catechol- (EC, ECG) and pyrogallol-type catechins (EGC, EGCG). Theaflavins are classified into four types: theaflavin (TF), theaflavin-3-gallate (TF3G), theaflavin-3′-gallate (TF3′G), and theaflavin-3,3′-digallate (TFDG). Teadenol A (TA) and teadenol B (TB) are catechin derivatives specifically detected in post-fermented teas produced through fermentation by *Aspergillus* species, with TA derived from EGCG and TB from GCG^26^. In this study, we investigated whether catechins inhibit the formation of the spike RBD-ACE2 complex using a newly developed cell-based pseudovirus assay system. Furthermore, we analyzed the structure–activity relationships (SARs) of structurally diverse catechins to identify the key molecular features responsible for their antiviral effects.

## Results

### Establishment of a cell-based SARS-CoV-2 infection assay

**Fig. 2:**
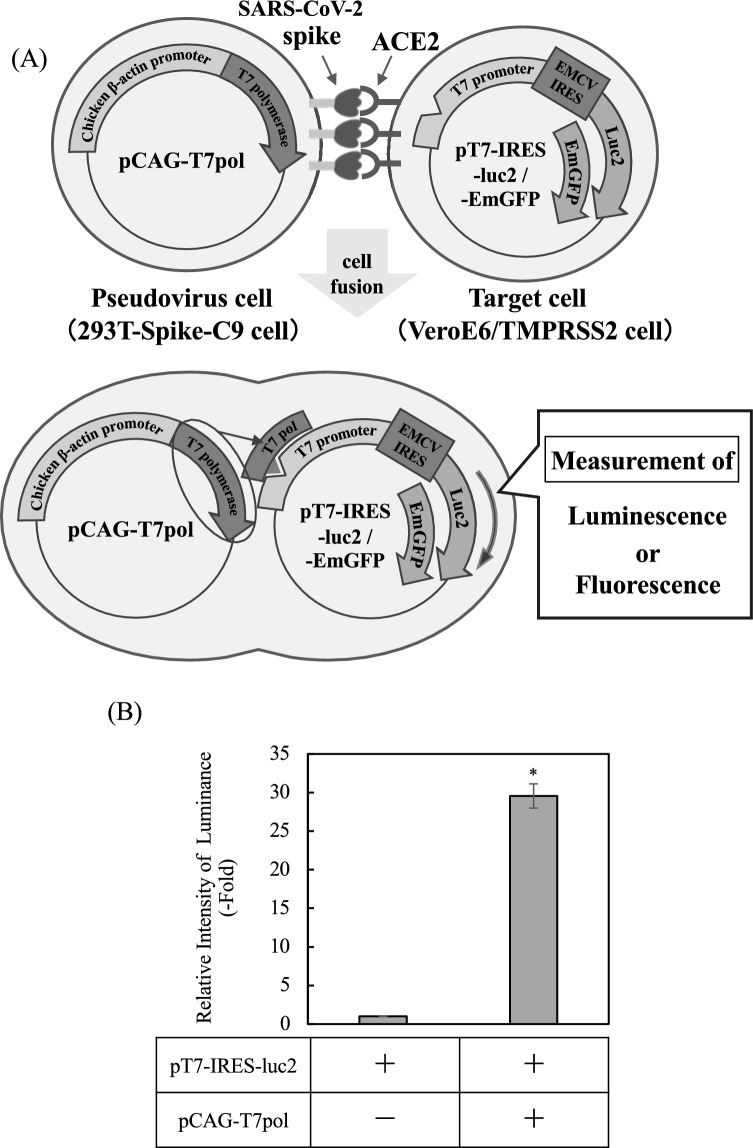
Overview of the cell-based severe acute respiratory syndrome coronavirus 2 (SARS-CoV-2) infection assay. We established a pseudovirus infection assay system on the basis of cell fusion induced by the interaction between the SARS-CoV-2 spike protein receptor-binding domain (RBD) and angiotensin-converting enzyme 2 (ACE2). The overall scheme is illustrated in (**A**). Cells expressing the SARS-CoV-2 spike protein and T7 polymerase were used as pseudovirus cells, while target cells were engineered to express ACE2 and carry a luciferase reporter gene (Luc2) under the regulation of the T7 promoter. Upon binding of the spike RBD to ACE2 and subsequent cell fusion, T7 polymerase from the pseudovirus cells activates the expression of Luc2 in the target cells. By adding the luciferase substrate, infection can be quantitatively measured on the basis of luminescence intensity. The quantification of luciferase activity in 293 T cells co-transfected with pT7-IRES-Luc2 and pCAG-T7pol is shown in (**B**). Detailed experimental procedures are described in the Methods. Luciferase activity is shown as a fold increase compared with control cells lacking pCAG-T7pol transfection. Data are presented as means ± standard deviations (SD) of four independent experiments, and were analyzed using Student’s t-test (*P < 0.001).

**Fig. 3:**
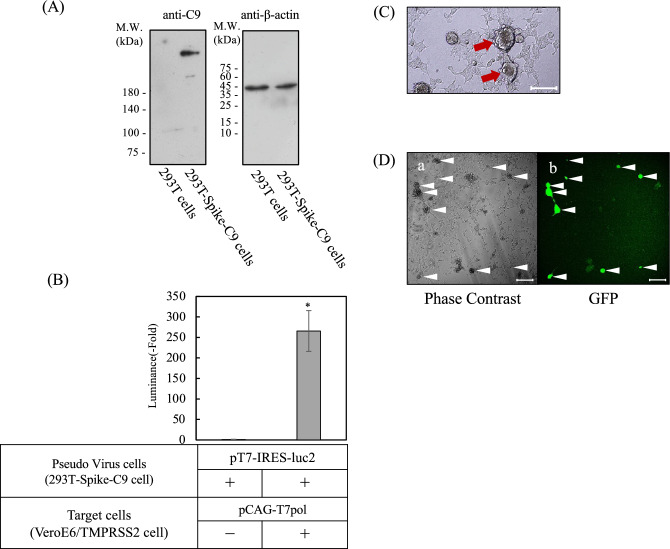
Establishment of the cell-based severe acute respiratory syndrome coronavirus 2 (SARS-CoV-2) infection assay. (**A**) Expression of the spike protein in the established 293 T-Spike-C9 cells was confirmed by western blotting using an anti-rhodopsin antibody targeting the C9 peptide fused to the C-terminus of the spike protein. β-actin was detected as a loading control using an anti-β-actin antibody. Details are described in the Methods. Original images of full-length blots (X-ray film scans) are provided in the Supplemental Fig. S2. (**B**) Pseudovirus cells were co-cultured with target cells for 20 h, and Luc2 expression was quantified. Luciferase activity is shown as the fold increase relative to the control, where mock pseudovirus cells (not transfected with pCAG-T7) were co-cultured with target cells. Data are presented as means ± standard deviations (SD) of four independent experiments, and were analyzed using Student’s t-test (*P < 0.001). (**C**) Microscopic image captured after 20 h co-culture with pseudovirus and target cells. Arrows indicate large multinucleated cells formed by cell fusion. (**D**) Fluorescence and phase contrast images following co-culture of pseudovirus and target cells in which the reporter plasmid was changed from pT7-IRES-Luc2 to pT7-IRES-EmGFP. *a* shows the phase contrast image, and *b* shows the corresponding green fluorescent protein (GFP) fluorescence. Arrowheads indicate large GFP-positive cells. The scale bars in (**C**) and (**D**) represent 100 µm.

Several polyphenols, including catechins and curcumin, have been proposed as potential inhibitors of SARS-CoV-2 infection^21^. The present study was undertaken to evaluate the antiviral potential of catechins. To this end, a cell-based SARS-CoV-2 infection assay was developed. This assay uses a cell–cell fusion mechanism that is mediated by the spike protein of the virus. An overview of the assay system is shown in Fig. 2A. In this system, 293 T cells expressing the SARS-CoV-2 spike protein were transfected with a T7 polymerase expression vector (pCAG-T7pol) to create pseudovirus cells. The target cells were generated by transfecting VeroE6/TMPRSS2 cells with the pT7-IRES-Luc2 vector, which enabled luciferase expression under the control of a T7 promoter. The interaction between spike-expressing pseudovirus cells and ACE2-expressing target cells leads to cell fusion, enabling T7 polymerase to initiate luciferase expression in the target cells. Luminescence intensity has been shown to serve as a quantitative indicator of infection. The initial step in validating the experimental system involved co-transfecting 293 T cells with pT7-IRES-luc2 and pCAG-T7pol and measuring luciferase luminescence. Increased luciferase activity was observed in transfected cells compared with control cells devoid of T7 polymerase (Fig. 2B). This finding serves as a validation of the T7 promoter-dependent reporter system. The establishment of a cell–cell interaction-based experimental system was achieved by generating 293 T cells that stably express the SARS-CoV-2 spike protein (293 T-Spike-C9 cells). Expression of the spike protein was confirmed by western blotting (Fig. 3A). Subsequently, cells were subjected to transfection with pT7-IRES-Luc2 to generate pseudovirus cells. These cells were then co-cultured with target cells to assess the efficiency of infection. After co-culture for 20 h, luminescence intensity was approximately 260-fold higher than that in the control, which included mock-transfected pseudovirus cells (Fig. 3B). Microscopic analysis revealed the presence of large multinucleated cells, indicating spike-ACE2-mediated cell fusion (Fig. 3C, red arrow). To verify that the fused cells were the result of a spike-ACE2 interaction, the experiment was repeated using the pT7-IRES-EmGFP reporter instead of the pT7-IRES-Luc2 reporter. The reporter drives expression of the fluorescent protein EmGFP in the presence of T7 polymerase. Strong green fluorescent protein (GFP) fluorescence was observed in multinucleated cells (Fig. 3D, white arrowhead). This observation confirms that fusion was dependent on the interaction between spike and ACE2. Collectively, these results substantiate the reliability of our assay in recapitulating spike-mediated infection and facilitating quantitative assessment of inhibitory compounds.

### Inhibitory effects of catechins in a cell-based SARS-CoV-2 infection assay

**Fig. 4:**
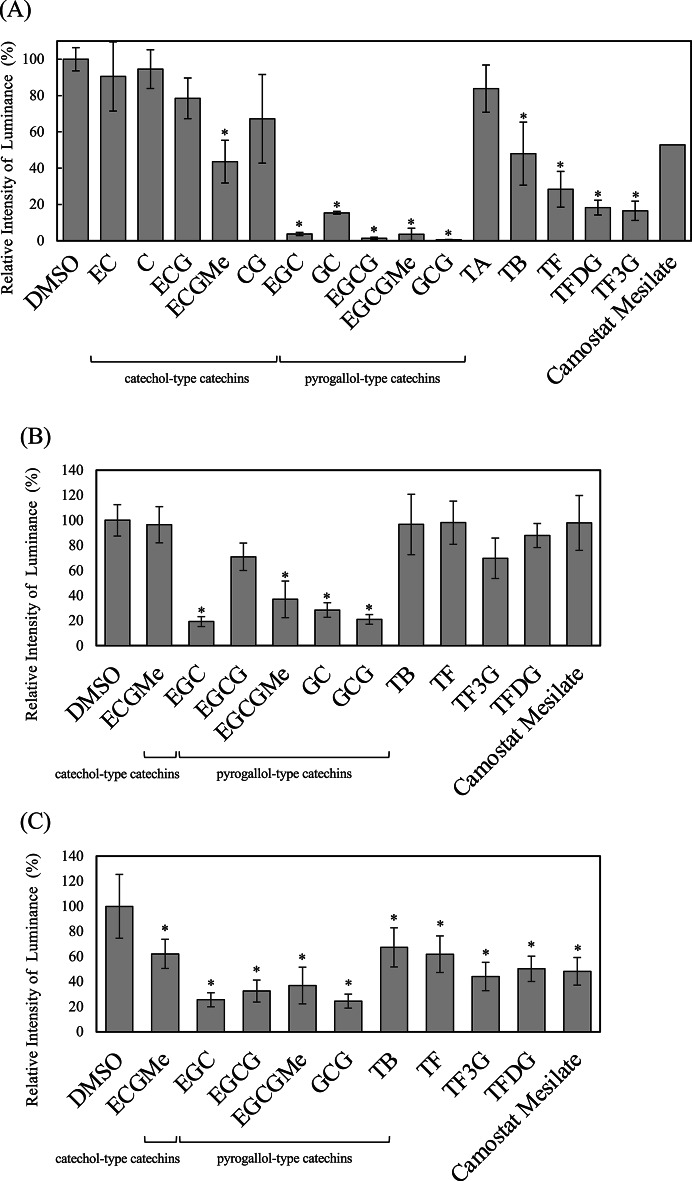
Inhibitory effects of catechins on severe acute respiratory syndrome coronavirus 2 (SARS-CoV-2) infection using a cell-based assay. (**A**) Pseudovirus cells and target cells were each incubated with catechins (100 µM) or vehicle (DMSO) for 30 min, followed by co-culture (final concentration: 50 µM) for 20 h in the continued presence of catechins to evaluate their inhibitory effect on viral infection. (**B**) Pseudovirus cells were pre-incubated with 100 µM catechins for 30 min. Subsequently, these cells were mixed with an equal volume of untreated target cells and co-cultured for 20 h, resulting in a final catechin concentration of 50 µM to assess the effects of catechins on the spike protein. (**C**) To evaluate the effects of catechins on target cells, including angiotensin-converting enzyme 2 (ACE2) and transmembrane serine protease 2 (TMPRSS2), target cells were pre-incubated with 100 µM catechins for 30 min. These cells were then mixed with an equal volume of untreated pseudovirus cells and co-cultured for 20 h (final concentration 50 µM). Data are presented as relative luciferase activity, with the vehicle control (DMSO) set to 100%. Data are presented as means ± standard deviations (SD) of four independent experiments, and were compared using a one-way analysis of variation (ANOVA) followed by Dunnett’s *post-hoc* test (*P < 0.001).

Using the established cell-based infection assay, we evaluated the inhibitory effects of 15 catechin derivatives (C, CG, GC, GCG, EC, ECG, EGC, EGCG, ECGMe, EGCGMe, TF, TF3G, TFDG, TA, and TB). Pseudovirus and target cells were pre-incubated with each catechin (100 µM) for 30 min at 37 °C, followed by co-culture in the continued presence of catechins for 20 h. Camostat mesylate was utilized as a control to assess the efficacy of potential infection inhibitors. The initial phase of the SARS-CoV-2 infection is marked by the binding of the spike protein RBD to ACE2, which is followed by viral entry via two distinct pathways: the TMPRSS2-mediated membrane fusion pathway and the TMPRSS2-independent endocytic pathway. Camostat mesylate selectively inhibits the former pathway by targeting TMPRSS2 and led to 50% inhibition of the infection (Fig. 4A). Luminescence analysis revealed that nine catechins (GCG, EGCG, EGCGMe, EGC, GC, TF3G, TFDG, TF, and ECGMe) and TB significantly reduced luciferase activity relative to the vehicle control (dimethyl sulfoxide), indicating strong inhibition of cell–cell fusion between pseudovirus cells and target cells (Fig. 4A). In particular, five pyrogallol-type catechins (EGC, GC, EGCG, EGCGMe, and GCG) showed significant inhibition of cell–cell fusion between pseudovirus cells and target cells, suggesting a potential for these catechins to impede SARS-CoV-2 infection. To ascertain whether this inhibition targeted the spike protein or ACE2, we pre-incubated catechins either with pseudovirus cells or target cells separately before co-culturing with the other cell type. Interestingly, camostat mesylate, which is known to inhibit TMPRSS2 activity in target cells, inhibited infection only when pre-incubated with the target cells, demonstrating approximately 50% inhibition (Fig. 4C). By contrast, pre-incubating pseudovirus cells with camostat mesylate had little to no effect on infection (Fig. 4B). Camostat mesylate’s dependence on target cell pre-incubation for efficacy aligns with its known mechanism of action as a TMPRSS2 inhibitor acting on host cells. This demonstrates that the assay system accurately reflects the inhibitor’s mode of action, thereby confirming its effectiveness and appropriateness. When treating pseudovirus cells only, GCG, EGCGMe, GC, and EGC demonstrated significant inhibitory effects (Fig. 4B). However, these catechins and TB showed significant inhibitory effects when used to treat target cells only (Fig. 4C). These results suggest that catechins act on both viral and host cell components, including the spike protein and ACE2.

Several studies have reported direct interactions between catechins and the spike protein RBD^27^. The present study focused on these interactions to identify the structural features of catechins that are critical for inhibitory activity. A subsequent analysis focusing on the B-ring structure revealed that catechins with a catechol-type configuration bearing hydroxyl groups at the 3- and 4-positions, such as C, EC, CG, and ECG, did not demonstrate significant inhibition. However, pyrogallol-type catechins with an additional hydroxyl group at the 5-position, such as EGC, GC, GCG, and EGCG, exhibited significant inhibitory activity (Fig. 4A). Notably, the four catechins that interacted with the spike protein (EGCGMe, GCG, GC, and EGC) all share a common feature: a pyrogallol-type B-ring structure (Figs. 1A and 4B). These findings indicate that the pyrogallol-type B-ring is a critical structural determinant for catechin-mediated inhibition of SARS-CoV-2 infection. A comparison between epi- and non-epi-type catechins, which differ in stereochemistry at the C2 position of the C-ring (Fig. 1A), revealed that both EGCG, which are important catechins found in green tea, effectively inhibited infection when pre-incubated with either spike-expressing pseudovirus cells or ACE2-expressing target cells (Fig. 4A). However, when incubated with spike protein alone, only GCG exhibited significant inhibition (Fig. 4B), suggesting that the non-epi configuration may facilitate more effective interaction with the spike protein. We also evaluated cell viability using PI staining. Consistent with previous studies^27^, catechins did not exhibit severe cytotoxicity toward 293 T-Spike-C9 cells (Supplementary Fig. S1). In contrast, when 293 T-Spike-C9 cells were treated with catechins in a suspended state, we observed non-specific inhibition of cell adhesion to the culture dish. It is possible that catechins broadly bind to the membrane proteins including spike proteins expressed on the cell surface, thereby interfering with a wide range of cellular functions. Since the potent antiviral effect observed in our infection assay (Fig. 4B) is structurally selective (i.e., pyrogallol-type selective) and the luciferase assay specifically monitors spike-mediated cell fusion, this potent and selective antiviral activity is clearly distinct from the non-specific adhesion impairment seen in suspension.

### Docking simulation of catechins with spike RBD

**Fig. 5:**
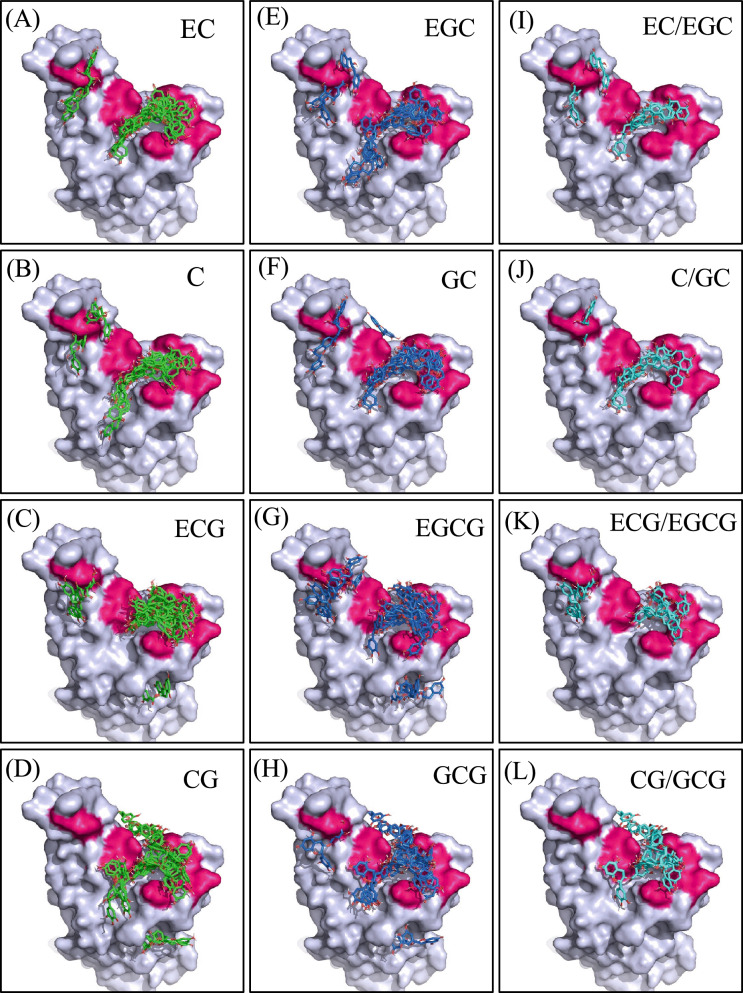
Docking models of catechins with the severe acute respiratory syndrome coronavirus 2 (SARS-CoV-2) spike receptor-binding domain (RBD). Panels (**A**)–(**D**) show the superimposed docking poses of catechol-type catechins (epicatechin [EC], catechin [C], epicatechin gallate [ECG], catechin gallate [CG]) with the SARS-CoV-2 spike RBD, while panels (**E**)–(**H**) show the corresponding pyrogallol-type catechins (epigallocatechin [EGC], gallocatechin [GC], epigallocatechin gallate [EGCG], gallocatechin gallate [GCG]). Panels (**I**)–(**L**) illustrate the representative binding modes shared between each catechol-type catechin and its pyrogallol-type counterpart to the SARS-CoV-2 spike RBD. The calculated binding affinities of these pairs exhibiting common binding modes are summarized in Table 1. The complex structures of the SARS-CoV-2 spike RBD and the angiotensin-converting enzyme 2 (ACE2) receptor were obtained from the Protein Data Bank. The three-dimensional structures of all catechins were retrieved from PubChem. After structural optimization, docking simulations were conducted using AutoDock Vina. Detailed experiments are described in the Methods section.

**Fig. 6:**
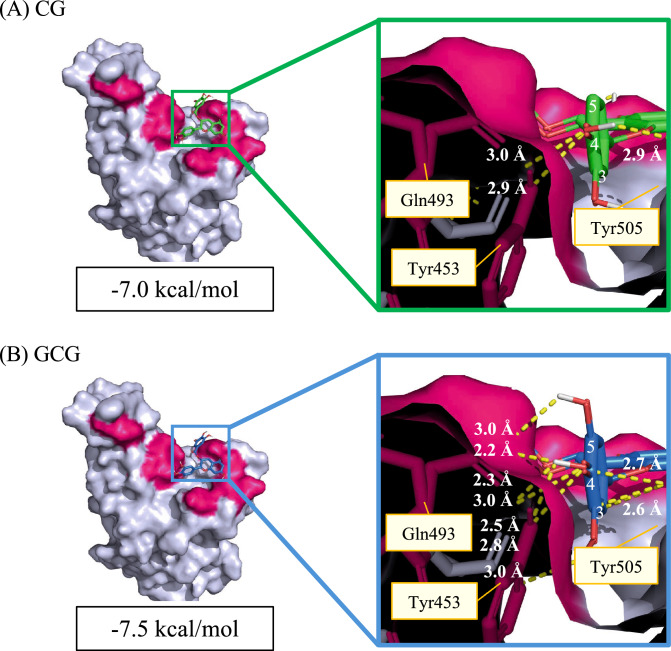
Detailed comparison of CG and GCG binding to the spike receptor-binding domain (RBD) in the common binding mode. Docking mode 4 (Table 1) was identified as a shared binding conformation between (**A**) catechin gallate (CG) and (**B**) gallocatechin gallate (GCG) and was visualized using PyMOL to illustrate the hydrogen bonding patterns with the severe acute respiratory syndrome coronavirus 2 (SARS-CoV-2) spike RBD. The red regions of the catechin structures indicate the oxygen atoms at positions 3, 4, and 5 of the B-ring (only 3 and 4 in the case of CG). Yellow dashed lines represent the predicted hydrogen bonds between the oxygens of catechins and key residues on the spike RBD (Gln493, Tyr453, and Tyr505), indicating stable interactions within the common binding pocket. Interatomic distances for these hydrogen bonds (≦3.0 Å) are also shown, with shorter distances suggesting stronger interactions. The docking scores for CG and GCG in this mode were − 7.0 and − 7.5 kcal/mol, respectively, indicating that GCG showed stronger binding affinity than CG in the same binding conformation.

In silico docking simulations of selected catechins with the RBD of the SARS-CoV-2 spike protein were performed using AutoDock Vina to elucidate the molecular basis for the observed inhibition. The docking poses of both catechol-type (A–D, green) and pyrogallol-type (E–H, blue) catechins are shown in Fig. 5. The pink regions represent 10 key amino acid residues predicted in silico to be critical for the interaction between the RBD and ACE2^28^. The importance of three of these amino acid residues for actual binding was confirmed by in vitro pull-down assays using recombinant RBD and ACE2^22^. A remarkable observation of the present study is that all catechins were shown to bind to the cavity of the RBD located around the critical amino acids for binding to ACE2 (Fig. 5), which occurred in a variety of binding modes. The results of these docking simulations were analyzed to extract catechin pairs that share the same binding mode by comparing catechol-type molecules (EC, C, ECG, CG) with their corresponding pyrogallol-type counterparts (EGC, GC, EGCG, GCG; Fig. 5I–L, light blue). The energies for these dockings, which share the same binding mode, are presented in Table 1. The pyrogallol-type catechins exhibited stronger binding (lower docking energies) than the catechol-type catechins (Table 1). These results indicate that pyrogallol-type catechins, despite having equivalent binding modes, exhibit an increased affinity for the RBD compared with their catechol-type counterparts. Binding mode 4, which is listed in Table 1 as a common binding conformation for CG and GCG, is shown in an enlarged view in Fig. 6. In GCG, the oxygen at the 5-position of the B-ring forms a hydrogen bond with Gln493 (3.0 Å; Fig. 6B), whereas CG lacks a hydroxyl group at this position and therefore cannot establish the same interaction (Fig. 6A). The presence of an additional hydrogen bond in GCG appears to have a significant impact on molecular orientation, thereby facilitating the formation of a new hydrogen bond between the hydroxyl group at the 3-position of the B-ring and the oxygen atom of Tyr453, with a distance of 3.0 Å. Additionally, a notable shortening in distance is observed among the hydrogen bonds involving the oxygen and hydroxyl group at the 4-position of the B-ring, suggesting greater strength in these interactions. These effects synergistically enhance the overall binding strength, and findings indicate that the presence of a hydroxyl group at the 5-position of the B-ring significantly enhances the interaction with the RBD, even though binding occurs within the same region. The stereoisomeric nature of epi- and non-epi-type catechins means that their three-dimensional structures exhibit significant differences, which complicates the identification of a shared binding mode. Binding energies for the catechins with the highest values obtained in docking simulations are presented in Table 2. Overall, non-epi-type catechins (C, CG, GC, GCG) exhibited stronger binding to the spike protein RBD than their epi-type counterparts (EC, ECG, EGC, EGCG). This phenomenon can be attributed to the transconfiguration of non-epi-type catechins, which allows for an increased contact area with the protein surface and positions the hydroxyl group at the 5-position of the B-ring in a favorable orientation for binding. This, in turn, enhances inhibitory activity. Collectively, the docking simulation results support our in vitro findings, demonstrating that catechins with pyrogallol-type B-rings and non-epicatechin stereochemistry exhibit stronger and more versatile binding to the spike RBD than their catechol-type counterparts. These structural features are likely to act synergistically to inhibit the RBD-ACE2 interaction.

**Table 1:** Comparison of binding affinity between catechol-type and pyrogallol-type catechins toward severe acute respiratory syndrome coronavirus 2 (SARS-CoV-2) spike receptor-binding domain (RBD) at the common binding mode.

Affinity (kcal/mol)								
Mode	EC	EGC	ECG	EGCG	C	GC	CG	GCG
1	− 6.2	− 6.2	− 7.1	− 7.4	− 6.5	− 6.6	− 7.4	− 7.8
2	− 6.0	− 6.1	− 7.1	− 7.1	− 6.4	− 6.5	− 6.9	− 7.8
3	− 6.0	− 6.0	− 6.9	− 7.0	− 6.0	− 6.4	− 6.9	− 7.8
4	− 6.0	− 6.0	− 6.6	− 7.0	− 5.9	− 6.1	− 7.0	− 7.5
5	− 6.0	− 6.0	− 6.6	− 6.7	− 5.6	− 5.7	− 6.7	− 7.5
6	− 5.6	− 5.9	− 6.8	− 6.6	− 5.4	− 5.7	− 6.7	− 7.5
7	− 5.8	− 5.9	–	–	− 5.4	− 5.5	− 6.8	− 7.3
8	− 6.2	− 5.7	–	–	–	–	− 6.6	− 7.2
9	− 5.6	− 5.7	–	–	–	–	− 6.6	− 7.0
10	− 5.5	− 5.6	–	–	–	–	− 6.4	− 7.0
11	–	–	–	–	–	–	− 6.4	− 6.8
Average	− 5.89	− 5.91	− 6.85	− 6.97	− 5.89	− 6.07	− 6.76	− 7.38

**Table 2:** Comparison of the five strongest binding modes between epicatechin-type and non-epicatechin-type catechins.

Affinity (kcal/mol)								
Mode	EC	C	ECG	CG	EGC	GC	EGCG	GCG
1	− 6.5	− 6.5	− 7.1	− 7.4	− 6.3	− 6.6	− 7.4	− 7.8
2	− 6.4	− 6.5	− 7.1	− 7.4	− 6.2	− 6.6	− 7.4	− 7.8
3	− 6.2	− 6.4	− 6.9	− 7.0	− 6.1	− 6.5	− 7.3	− 7.5
4	− 6.2	− 6.1	− 6.8	− 6.9	− 6.1	− 6.4	− 7.1	− 7.5
5	− 6.1	− 6.1	− 6.8	− 6.9	− 6.0	− 6.4	− 7.1	− 7.5
Average	− 6.28	− 6.32	− 6.94	− 7.12	− 6.14	− 6.50	− 7.26	− 7.62

## Discussion

In this study, we investigated the inhibitory effects of structurally diverse catechins on the interaction between the SARS-CoV-2 spike protein RBD and the human ACE2 receptor using a cell-based pseudovirus assay. Our findings clearly demonstrate that catechins with a pyrogallol-type B-ring exhibit significantly greater inhibitory activity than their catechol-type counterparts. Additionally, it is thought that the non-epi stereochemistry may also contribute to these inhibitory effects to some extent. These conclusions were consistently supported by both experimental infection assays and in silico docking simulations. Previous computational studies have identified amino acid residues within the RBD (specifically Tyr449, Tyr453, Leu455, Asn487, Tyr489, Gln493, Gln498, Thr500, Asn501, and Tyr505) as being critical for interactions with ACE2^22^. In this study, the importance of Tyr453, Gln493, and Asn501 was experimentally validated by performing pull-down assays. The docking simulations showed that a significant proportion of the catechins exhibited a binding preference for regions close to these residues (Fig. 5, pink), confirming their ability to inhibit RBD-ACE2 complex formation. Whereas prior studies have primarily focused on EGCG because of its abundance in green tea, our study highlights that pyrogallol-type (GC, GCG, EGC, and EGCGMe) and non-epi-type catechins (GCG) may offer superior inhibition of SARS-CoV-2 entry. This study represents the first detailed SAR analysis of catechins, specifically focusing on the comparative potency related to B-ring hydroxylation and C-ring stereochemistry.

To understand how strong these binding affinities are, we performed additional docking simulations using two control antiviral drugs: simeprevir and remdesivir. Simeprevir is an approved hepatitis C virus protease inhibitor that, through drug repositioning studies, has been suggested to bind to the SARS-CoV-2 RBD and inhibit the Spike-ACE2 interaction^28,29^. Remdesivir is approved by the US Food and Drug Administration for the treatment of COVID-19. Its primary mechanism involves inhibiting the viral RNA-dependent RNA polymerase (RdRp)^30,31^, although some in silico studies have also suggested that it may bind to the RBD^32^. The results of docking analyses for these controls, performed using the identical protocol as our catechins, are presented in Supplementary Table S2. The average affinity for simeprevir was − 7.98 kcal/mol. The affinity of GCG, the most potent catechin in this study (average affinity: − 7.38 kcal/mol), was comparable to that of simeprevir. In contrast, remdesivir’s affinity was significantly lower (average affinity: − 6.81 kcal/mol), and was similar to that of the catechol-type catechin CG (average affinity: − 6.76 kcal/mol). These data confirm that the binding affinity of pyrogallol-type catechins to the RBD is potent, specific, and comparable to that of other dedicated RBD binders, rather than being a non-specific interaction observed with drugs targeting other viral proteins such as RdRp.

Notably, catechin epimerization occurs during tea processing, resulting in increased levels of non-epi-type catechins in ready-to-drink bottled teas^24^. Although these epimers are present in lower concentrations in freshly brewed tea, they may contribute substantially to the efficacy of the antiviral properties of tea. The present study, which focused on the RBD-ACE2 interaction, complements the existing body of research on catechins and related flavonoids against SARS-CoV-2. Previous studies have primarily focused on EGCG, attributing its antiviral effects to multiple mechanisms, including the inhibition of the main protease (Mpro) or papain-like protease (PLpro)^20,33^, or binding to heparan sulfate on host cells^12,19^. Other flavonoids, such as quercetin and myricetin, have also been reported to inhibit Mpro^34^. Although some studies report EGCG inhibiting Spike-ACE2 binding^12,35^, the present study is the first to conduct a detailed structure–activity relationship analysis comparing pyrogallol-type and catechol-type catechins, as well as epi- and non-epi-type stereochemistry, specifically for this mechanism. Our findings highlight that GCG (a non-epi, pyrogallol-type catechin) may offer superior inhibition of viral entry via this pathway, a finding that was not predictable from the EGCG-centric literature.

Several limitations of this study must be acknowledged. First, these findings are based on a pseudovirus cell–cell fusion assay. While this system (Figs. 2 and 3) effectively models the Spike-ACE2-mediated entry step, it does not utilize live SARS-CoV-2, nor does it account for other stages of the viral life cycle. Second, the observed inhibitory effects are based on in vitro experiments using cell lines (293 T and VeroE6/TMPRSS2), which may not fully reflect the complex cellular environment of primary human respiratory epithelial cells.

Finally, the practical application of these findings must be considered. The bioavailability of catechins, particularly EGCG, is known to be very low, with peak plasma concentrations rarely exceeding 1 µM^27,35^. The concentrations required for in vitro inhibition in our assay (100 µM) are thus unlikely to be achieved systemically. However, SARS-CoV-2 infection is primarily initiated at mucosal surfaces in the upper respiratory tract (oral and nasal cavity)^36^. Green tea beverages contain catechin concentrations (approximately 100 µM)^23–25^ similar to those effective in our assay. This suggests that localized mucosal delivery, rather than systemic administration, is a more viable therapeutic strategy. Frequent application via gargling, nasal sprays, or lozenges could maintain high local concentrations of pyrogallol-type catechins at the initial sites of infection, potentially offering a localized preventative or early therapeutic effect. A critical aspect of antiviral therapeutic development is the potential for reduced efficacy associated with viral evolution. Emerging variants of concern, such as Omicron (e.g., BA.1), harbor numerous mutations in the spike RBD that enhance ACE2 binding and confer immune evasion^36^. Notably, the Omicron BA.1 variant contains mutations at several key residues within the ACE2-binding interface, including Q493R, Q498R, and N501Y. The Q493R mutation is directly relevant to our findings, as our model (Fig. 6B) identified Gln493 as a critical hydrogen bond partner for the pyrogallol B-ring of GCG. The substitution of the neutral glutamine residue with the bulkier, positively charged arginine would almost certainly disrupt this specific H-bond, potentially weakening the binding affinity of this pose. However, as our docking analysis demonstrated (Fig. 5), catechins bind to the RBD cavity in multiple, diverse, and overlapping modes, rather than relying on a single conformation. It is therefore plausible that other binding poses, perhaps stabilized by interactions with conserved residues like Tyr453 (which is not mutated in Omicron BA.1), may remain effective. Considering the propensity of the virus to undergo genetic mutations and the capacity of catechins to engage the RBD through multiple independent binding modes as suggested by our docking data, catechins have the potential to provide a robust antiviral mechanism that is weakly susceptible to evasion by single-point mutations. Further experimental validation using pseudoviruses of emerging variants is warranted to confirm this hypothesis.

## Methods

### Materials

Teadenols A and B were obtained from the Japanese post-fermented tea “Kippuku-cha” as previously described^26,37^. Catechins and theaflavins were purchased from Nagara Science Co. Ltd. (Gifu, Japan). Unless otherwise noted, other reagents were obtained from Wako Pure Chemical Industries (Osaka, Japan) and Nacalai Tesque (Kyoto, Japan). All reagents used were of the highest available analytical grade.

### Plasmid design

All primers used for plasmid construction are listed in Supplementary Table S1. Genes encoding Luc2 and EmGFP were amplified by polymerase chain reaction (PCR) from pGL4.23 (Promega, Madison, WI, USA) and pcDNA6.2/N-EmGFP-DEST (Thermo Fisher Scientific, Waltham, MA, USA), respectively, using the Luc2/EmGFP primer set. The pT7-IRES His-C vector (Takara Bio, Ohtsu, Japan) was linearized by PCR. These amplified fragments were then cloned into the linearized vector downstream of the internal ribosome entry site (IRES) using the In-Fusion cloning system (Takara Bio), resulting in the reporter plasmids pT7-IRES-Luc2 and pT7-IRES-EmGFP. The SARS-CoV-2 spike protein expression vector, pBA-spike-C9, was constructed by amplifying the spike gene (including a C-terminal C9 tag) from pcDNA3.1-SARS2-spike using the SARS-CoV2-Spike-C9 primer set. The pBApo-EF1α Pur vector (Takara Bio) was linearized by BamHI digestion. The amplified spike fragment was cloned into this linearized vector using the In-Fusion cloning system. The plasmid pcDNA3.1-SARS2-spike was a gift from Fang Li (Addgene plasmid #145032)^22^. The T7 RNA polymerase expression vector, pCAG-T7pol, was a gift from Dr. Ian Wickersham (Addgene plasmid #59926).

### Cell culture

VeroE6/TMPRSS2 cells and 293 T cells were maintained in Dulbecco’s Modified Eagle Medium (DMEM; Sigma, St. Louis, MO, USA) supplemented with 5% fetal bovine serum (FBS), penicillin, and streptomycin, at 37 °C in a humidified 5% CO_2_ incubator. VeroE6/TMPRSS2 cells (JCRB1819)^38^ were obtained from JCRB Cell Bank (Osaka, Japan). 293 T cells were obtained from RIKEN BRC through the National Bio-Resource Project of MEXT, Japan.

### Transfection of DNA

Transfections were performed using polyethyleneimine Max (PEI; Polysciences, Inc., Warrington, PA, USA) as previously described^39^. Briefly, 4 µg plasmid DNA was mixed with 250 µL Opti-MEM and incubated at 25 °C for 5 min. Separately, 10 µg PEI was diluted in 250 µL Opti-MEM and was also incubated for 5 min. The PEI and DNA solutions were then combined and incubated for a further 20 min. The mixture was added to a six-well plate containing 5 × 10^5^ cells in 2 mL DMEM with 5% FBS. After 4 h, the medium was replaced with fresh DMEM containing 5% FBS and the cells were incubated overnight. To generate spike-expressing cells, 293 T cells were transfected with pBA-Spike-C9 and selected with 2 µg/mL puromycin. Clonal selection was performed by limiting dilution. Clones expressing high levels of spike protein were identified by western blotting, as previously described^39^ using an anti-rhodopsin antibody (Rho 1D4, Sigma) recognizing the C9 tag. β-actin was used as a loading control.

### Cell-based SARS-CoV-2 infection assay

Pseudovirus cells were generated by transfecting 293 T-Spike-C9 cells with pCAG-T7pol using PEI as described above. Target cells were generated by transfecting VeroE6/TMPRSS2 cells with pT7-IRES-Luc2 (or pT7-IRES-EmGFP for fluorescence assays). VeroE6/TMPRSS2 cells highly express ACE2, the receptor for SARS-CoV-2 entry^40,41^. Both cell types were washed with saline and detached using TrypLE Express (Thermo Fisher Scientific) for 3 min at 37 °C. They were resuspended in DMEM with 5% FBS. Pseudovirus and target cells (2 × 10^4^ each) were pre-incubated with catechins (100 µM) or vehicle (DMSO) for 30 min, then co-seeded and co-cultured for 20 h at 37 °C (final concentration: 50 µM). The next day, luciferase activity was quantified using the PicaGene™ luciferase assay system (Toyo Ink, Tokyo, Japan), and luminescence was measured with a Luminoskan™ Ascent (Thermo Fisher Scientific). Data are presented as means ± standard deviations (SD) of four independent experiments.

### Docking simulation

The crystal structure of the SARS-CoV-2 RBD-ACE2 complex (PDB ID: 6M0J) was downloaded from the Protein Data Bank (https://www.rcsb.org/)^42^. Using PyMOL^43^, all water molecules, ligands, ions, and the ACE2 portion were removed. Polar hydrogens and charges were added in AutoDock Vina (version 1.5.7, https://ccsb.scripps.edu/mgltools/)^44^, and the structure was converted to PDBQT format. In accordance with previous studies, 10 RBD residues critical for ACE2 interaction (Tyr449, Tyr453, Leu455, Asn487, Tyr489, Gln493, Gln498, Thr500, Asn501, and Tyr505)^28,40^ were designated as flexible side chains. Separate PDBQT files were prepared for flexible and rigid regions. Catechin structures were obtained from PubChem (https://pubchem.ncbi.nlm.nih.gov)^45^ in SDF format, energy-minimized using the Merck Molecular Force Field 94 (MMFF94)^46^, and converted to PDBQT format. The grid box was configured to encompass the entire RBD. Docking simulations were conducted in AutoDock Vina by assigning each catechin as a ligand with rotatable bonds, following standard procedures^21,47,48^ in Open Babel^46,49,50^ and converted to PDBQT format. The specific grid box parameters used to encompass the entire ACE2-binding interface (RBD) were the center coordinates (x = − 32.257, y = 30.241, z = 1.335) and dimensions (size x = 40, y = 50, z = 30). No constraints were applied during the docking run, and the Vina parameters were set as follows: seed = 827,301,009, cpu = 8, exhaustiveness = 32, num_modes = 100, and energy range = 5.

## Supplementary Information

Supplementary Information 1.

Supplementary Information 2.

Supplementary Information 3.

Supplementary Information 4.

## Data Availability

The datasets generated and analyzed during the current study are available from the corresponding author on reasonable request. All primary docking data (input and output files) used for computational analysis are available upon request to the corresponding author for the purposes of reproducibility and replication.

## Abbreviations

CH: (−)-Catechin hydrate
EC: (−)-Epicatechin
CG: (−)-Catechin gallate
ECG: (−)-Epicatechin gallate
ECGMe: (−)-Epicatechin 3-(3″-O-methyl) gallate
GC: (−)-Gallocatechin
EGC: (−)-Epigallocatechin
GCG: (−)-Gallocatechin gallate
EGCG: (−)-Epigallocatechin gallate
EGCGMe: (−)-Epigallocatechin 3-(3″-O-methyl) gallate
TF: Theaflavin
TF3G: Theaflavin 3-gallate
TFDG: Theaflavin 3,3′-di-O-gallate
TA: Teadenol A
TB: Teadenol B
ACE2: Angiotensin-converting enzyme 2
RBD: Receptor-binding domain

## References

[1] Hoffmann, M. et al. SARS-CoV-2 cell entry depends on ACE2 and TMPRSS2 and is blocked by a clinically proven protease inhibitor. *Cell* **181**, 271-280.e8 (2020). 32142651 10.1016/j.cell.2020.02.052 PMC7102627

[2] Shang, J. et al. Cell entry mechanisms of SARS-CoV-2. *Proc. Natl. Acad. Sci. U. S. A.* **117**, 11727–11734 (2020). 32376634 10.1073/pnas.2003138117 PMC7260975

[3] Stäb, S. et al. Up, up, down, down: The structural biology of the SARS-CoV-2 spike protein and how it cheats the immune system. *Crystallogr. Rev.* **30**, 74–117 (2024).

[4] Jackson, C. B., Farzan, M., Chen, B. & Choe, H. Mechanisms of SARS-CoV-2 entry into cells. *Nat. Rev. Mol. Cell Biol.* **23**, 3–20 (2021). 34611326 10.1038/s41580-021-00418-x PMC8491763

[5] Isaacs, C. E. et al. Digallate dimers of (−)-epigallocatechin gallate inactivate herpes simplex virus. *Antimicrob. Agents Chemother.* **55**, 5646–5653 (2011). 21947401 10.1128/AAC.05531-11 PMC3232753

[6] Huang, H. C. et al. (−)-Epigallocatechin-3-gallate inhibits entry of hepatitis B virus into hepatocytes. *Antivir. Res.* **111**, 100–111 (2014). 25260897 10.1016/j.antiviral.2014.09.009

[7] Colpitts, C. C. & Schang, L. M. A small molecule inhibits virion attachment to heparan sulfate- or sialic acid-containing glycans. *J. Virol.* **88**, 7806–7817 (2014). 24789779 10.1128/JVI.00896-14 PMC4097786

[8] Weber, C., Sliva, K., Von Rhein, C., Kümmerer, B. M. & Schnierle, B. S. The green tea catechin, epigallocatechin gallate inhibits chikungunya virus infection. *Antivir. Res.* **113**, 1–3 (2015). 25446334 10.1016/j.antiviral.2014.11.001

[9] Wu, C.-Y., Yu, Z.-Y., Chen, Y.-C. & Hung, S.-L. Effects of epigallocatechin-3-gallate and acyclovir on herpes simplex virus type 1 infection in oral epithelial cells. *J. Formos. Med. Assoc.* **120**, 2136–2143 (2021). 33390306 10.1016/j.jfma.2020.12.018

[10] Weber, J. M., Ruzindana-Umunyana, A., Imbeault, L. & Sircar, S. Inhibition of adenovirus infection and adenain by green tea catechins. *Antivir. Res.* **58**, 167–173 (2003). 12742577 10.1016/s0166-3542(02)00212-7

[11] Henss, L. et al. The green tea catechin epigallocatechin gallate inhibits SARS-CoV-2 infection. *J. Gen. Virol.* **102**, 1574 (2021). 10.1099/jgv.0.001574 PMC8290267 33830908

[12] LeBlanc, E. V. & Colpitts, C. C. The green tea catechin EGCG provides proof-of-concept for a pan-coronavirus attachment inhibitor. *Sci. Rep.* **12**, 12899 (2022). 35902713 10.1038/s41598-022-17088-0 PMC9330937

[13] Ohgitani, E. et al. Significant inactivation of sars-cov-2 in vitro by a green tea catechin, a catechin-derivative, and black tea galloylated theaflavins. *Molecules* **26**, 3572 (2021). 34208050 10.3390/molecules26123572 PMC8230566

[14] Ohgitani, E. et al. Rapid inactivation in vitro of sars-cov-2 in saliva by black tea and green tea. *Pathogens* **10**, 721 (2021). 34201131 10.3390/pathogens10060721 PMC8227886

[15] Umeda, M. et al. Preventive effects of tea and tea catechins against influenza and acute upper respiratory tract infections: A systematic review and meta-analysis. *Eur. J. Nutr.* **60**, 4189–4202 (2021). 34550452 10.1007/s00394-021-02681-2 PMC8456193

[16] Saeki, K. et al. In vitro and in silico studies of the molecular interactions of epigallocatechin-3-o-gallate (egcg) with proteins that explain the health benefits of green tea. *Molecules*10.3390/molecules23061295 (2018). 29843451 10.3390/molecules23061295 PMC6099932

[17] Šturm, L. et al. Interactions of (−)-epigallocatechin-3-gallate with model lipid membranes. *Biochim. Biophys. Acta* **1864**, 183999 (2022). 10.1016/j.bbamem.2022.183999 35820494

[18] Dai, T. et al. Analysis of inhibitory interaction between epigallocatechin gallate and alpha-glucosidase: A spectroscopy and molecular simulation study. *Spectrochim. Acta A Mol. Biomol. Spectrosc.* **230**, 118023 (2020). 31927512 10.1016/j.saa.2019.118023

[19] Revuelta, J. et al. Synthetic heparan sulfate mimics based on chitosan derivatives show broad-spectrum antiviral activity. *Commun. Biol.* **8**, 360 (2025). 40038521 10.1038/s42003-025-07763-z PMC11880534

[20] Kato, Y. et al. Tea catechins in green tea inhibit the activity of SARS-CoV-2 main protease via covalent adduction. *J. Agric. Food Chem.* **73**, 4116–4125 (2025). 39907399 10.1021/acs.jafc.4c11685

[21] Jena, A. B., Kanungo, N., Nayak, V., Chainy, G. B. N. & Dandapat, J. Catechin and curcumin interact with S protein of SARS-CoV2 and ACE2 of human cell membrane: Insights from computational studies. *Sci. Rep.* **11**, 2043 (2021). 33479401 10.1038/s41598-021-81462-7 PMC7820253

[22] Shang, J. et al. Structural basis of receptor recognition by SARS-CoV-2. *Nature* **581**, 221–224 (2020). 32225175 10.1038/s41586-020-2179-y PMC7328981

[23] Botten, D., Fugallo, G., Fraternali, F. & Molteni, C. Structural properties of green tea catechins. *J. Phys. Chem. B* **119**, 12860–12867 (2015). 26369298 10.1021/acs.jpcb.5b08737

[24] Seto, R., Nakamura, H., Nanjo, F. & Hara, Y. Preparation of epimers of tea catechins by heat treatment. *Biosci. Biotech. Biochcm* **61**, 1434–1439 (1997).

[25] Xu, J. Z., Leung, L. K., Huang, Y. & Chen, Z. Y. Epimerisation of tea polyphenols in tea drinks. *J. Sci. Food Agric.* **83**, 2412–2419 (2003).

[26] Wulandari, R. A. et al. New phenolic compounds from *Camellia sinensis* L. leaves fermented with *Aspergillus* sp. *J. Nat. Med.* **65**, 584–597 (2011). 10.1007/s11418-011-0515-0 21327519

[27] Jang, M. et al. Tea polyphenols EGCG and theaflavin inhibit the activity of SARS-CoV-2 3CL-protease in vitro. *Evid. Based Complement. Alternat. Med.* **2020**, 5630838 (2020). 32963564 10.1155/2020/5630838 PMC7499281

[28] Shahbazi, B., Mafakher, L. & Teimoori-Toolabi, L. Different compounds against Angiotensin-Converting Enzyme 2 (ACE2) receptor potentially containing the infectivity of SARS-CoV-2: An in silico study. *J. Mol. Model.* **28**, 82 (2022). 35249180 10.1007/s00894-022-05059-1 PMC8898033

[29] Muturi, E. et al. Effects of simeprevir on the replication of SARS-CoV-2 in vitro and in transgenic hACE2 mice. *Int. J. Antimicrob. Agents* **59**, 106499 (2021). 34929295 10.1016/j.ijantimicag.2021.106499 PMC8679493

[30] Behera, S., Mahapatra, N., Tripathy, C. & Pati, S. Drug repurposing for identification of potential inhibitors against SARS-CoV-2 spike receptor-binding domain: An in silico approach. *Indian J. Med. Res.* **153**, 132 (2021). 33818470 10.4103/ijmr.IJMR_1132_20 PMC8184087

[31] Kokic, G. et al. Mechanism of SARS-CoV-2 polymerase stalling by remdesivir. *Nat. Commun.* **12**, 1–7 (2021). 33436624 10.1038/s41467-020-20542-0 PMC7804290

[32] Navabshan, I. et al. Computational lock and key and dynamic trajectory analysis of natural biophors against COVID-19 spike protein to identify effective lead molecules. *Mol. Biotechnol.* **63**, 898 (2021). 34159564 10.1007/s12033-021-00358-z PMC8219180

[33] Chiou, W. C. et al. The inhibitory effects of PGG and EGCG against the SARS-CoV-2 3C-like protease. *Biochem. Biophys. Res. Commun.* **591**, 130–136 (2022). 33454058 10.1016/j.bbrc.2020.12.106 PMC7787066

[34] Abian, O. et al. Structural stability of SARS-CoV-2 3CLpro and identification of quercetin as an inhibitor by experimental screening. *Int. J. Biol. Macromol.* **164**, 1693–1703 (2020). 32745548 10.1016/j.ijbiomac.2020.07.235 PMC7395220

[35] Liu, J. et al. Epigallocatechin gallate from green tea effectively blocks infection of SARS-CoV-2 and new variants by inhibiting spike binding to ACE2 receptor. *Cell Biosci.* **11**, 168 (2021). 34461999 10.1186/s13578-021-00680-8 PMC8404181

[36] Lamers, M. M. & Haagmans, B. L. SARS-CoV-2 pathogenesis. *Nat. Rev. Microbiol.* **20**, 270–284 (2022). 35354968 10.1038/s41579-022-00713-0

[37] Nagasawa, T., Ishimaru, K., Higashiyama, S., Hama, Y. & Mitsutake, S. Teadenol A in microbial fermented tea acts as a novel ligand on GPR120 to increase GLP-1 secretion. *Food Funct.* **11**, 10534–10541 (2020). 33185223 10.1039/d0fo02442b

[38] Nao, N. et al. Consensus and variations in cell line specificity among human metapneumovirus strains. *PLoS ONE* **14**, e0215822 (2019). 31013314 10.1371/journal.pone.0215822 PMC6478314

[39] Nagasawa, T. et al. Phytosphingosine is a novel activator of GPR120. *J. Biochem.* **164**, 27–32 (2018). 29373685 10.1093/jb/mvy017

[40] Li, W. et al. Angiotensin-converting enzyme 2 is a functional receptor for the SARS coronavirus. *Nature* **426**, 450–454 (2003). 14647384 10.1038/nature02145 PMC7095016

[41] Drosten, C., Preiser, W., Günther, S., Schmitz, H. & Doerr, H. W. Severe acute respiratory syndrome: Identification of the etiological agent. *Trends Mol. Med.* **9**, 325–327 (2003). 12928032 10.1016/S1471-4914(03)00133-3 PMC7128529

[42] Berman, H. M. et al. The protein data bank. *Nucleic Acids Res.* **28**, 235–242 (2000). 10592235 10.1093/nar/28.1.235 PMC102472

[43] Delano, W. The PyMOL molecular graphics system. *CCP4 Newsl. Protein Crystallogr.* **40**, 8–16 (2002).

[44] Trott, O. & Olson, A. J. AutoDock Vina: Improving the speed and accuracy of docking with a new scoring function, efficient optimization and multithreading. *J. Comput. Chem.* **31**, 455 (2010). 19499576 10.1002/jcc.21334 PMC3041641

[45] Kim, S. et al. PubChem substance and compound databases. *Nucleic Acids Res.* **44**, D1202–D1213 (2016). 26400175 10.1093/nar/gkv951 PMC4702940

[46] Halgren, T. A. Performance of MMFF94*. *Scope, Parameterization, J. Comput. Chem.* **17**, 490–519 (1996).

[47] Akishino, M. et al. Red algae-derived isofloridoside activates the sweet taste receptor T1R2/T1R3. *Food Biosci.* **50**, 102146 (2022).

[48] Nagasawa, T. et al. The molecular mechanism of phytosphingosine binding to FFAR4/GPR120 differs from that of other fatty acids. *FEBS Open Bio.* **11**, 3081–3089 (2021). 34535977 10.1002/2211-5463.13301 PMC8564095

[49] O’Boyle, N. M. et al. Open Babel: An open chemical toolbox. *J. Cheminform.* **3**, 1–14 (2011). 21982300 10.1186/1758-2946-3-33 PMC3198950

[50] Halgren, T. A. M. M. F. F. V. I. MMFF94s option for energy minimization studies. *J. Comput. Chem.* **20**, 720–729 (1999). 34376030 10.1002/(SICI)1096-987X(199905)20:7<720::AID-JCC7>3.0.CO;2-X

