# Supplementary material for: Pyrogallol B-ring enhances catechin binding to the SARS-CoV-2 spike receptor-binding domain to inhibit interaction with ACE2
Source: Sci Rep. 2026 Feb 28;16:11413. doi: 10.1038/s41598-026-41170-6 (PMC13057188; doi:10.1038/s41598-026-41170-6)
Supplement: Supplementary file 1 — Supplementary Information 1. [file 41598_2026_41170_MOESM1_ESM.pdf]

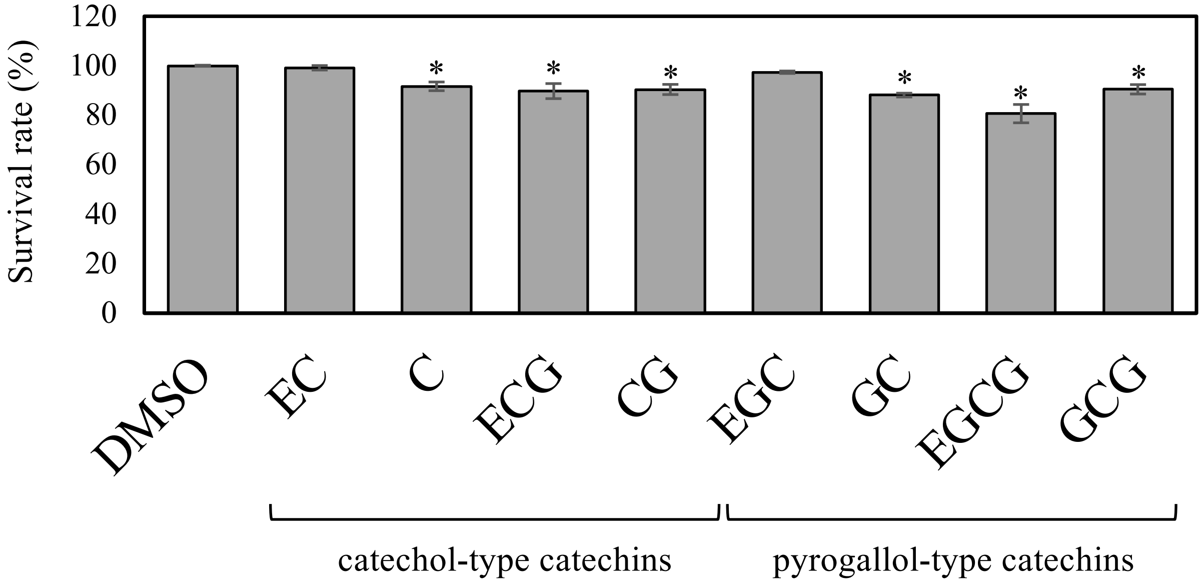

Supplementary Fig. S1. Assessment of cell viability of cells treated with catechins.
To rule out non-specific cytotoxicity, cell viability was evaluated using Propidium Iodide (PI) (Fujifilm Wako Pure Chemical Corporation, Osaka, Japan) staining. 293T-Spike-C9 cells were seeded at 2.5×10^4 cells/well. The cells were treated with each catechin derivative as the condition was equal to Fig. 4B; specifically, cells were incubated with 100 µM catechin for 30 min, followed by dilution to a final concentration of 50 µM and further incubation overnight at 37° C. The percentage of viable cells was quantified by flow cytometry using a MACSQuant analyzer (Miltenyi Biotec, Bergisch Gladbach, Germany) and analyzed with FlowJo software (FlowJo, LLC, Ashland, OR, USA). Data represent the mean ± SD of three independent experiments (n = 3). Statistical significance was determined using a one-way ANOVA followed by Dunnett's post-hoc test (* $P < 0.01$ vs. DMSO). Although some catechins showed a statistically significant reduction in viability compared to the DMSO control, the survival rates remained high (>80%), indicating that the potent antiviral effects observed in Figure 4 are not primarily attributable to cytotoxicity.

Supplemental Fig. S1 Matsumoto *et. al.*
