# Supplementary material for: Pyrogallol B-ring enhances catechin binding to the SARS-CoV-2 spike receptor-binding domain to inhibit interaction with ACE2
Source: Sci Rep. 2026 Feb 28;16:11413. doi: 10.1038/s41598-026-41170-6 (PMC13057188; doi:10.1038/s41598-026-41170-6)
Supplement: Supplementary file 2 — Supplementary Information 2. [file 41598_2026_41170_MOESM2_ESM.pdf]

(A)

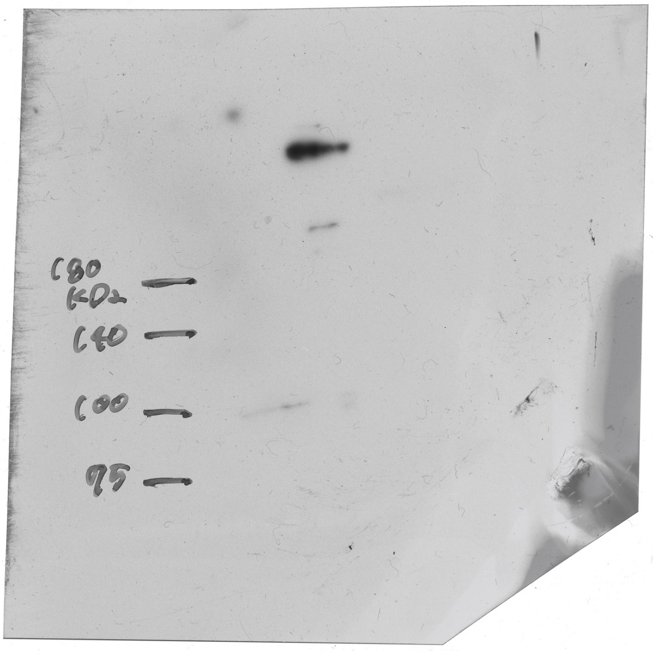

(B)

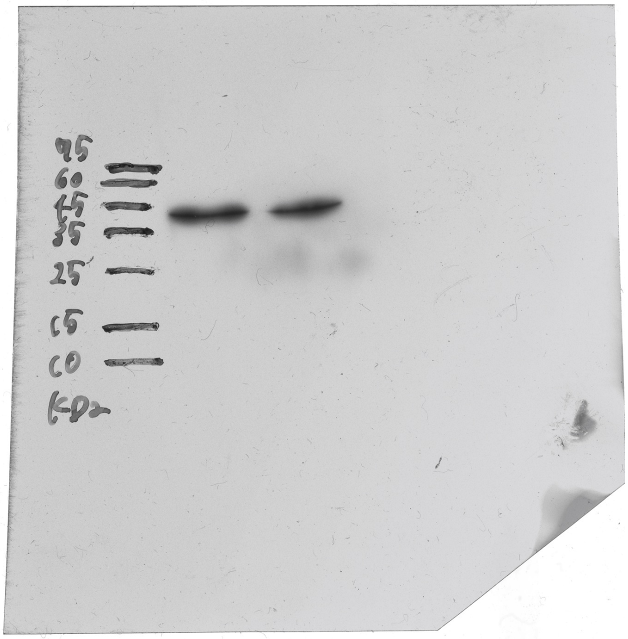

Supplemental Fig. S2. Original, uncropped images of Western blots for Figure 3A. (A) Full scan of the original X-ray film for the western blot using an anti-C9 antibody to detect the C9-tagged SARS-CoV-2 spike protein. (B) Full scan of the original X-ray film for the western blot using an anti-β-actin antibody as a loading control. The images represent the original, unprocessed data. The molecular weight marker positions were hand-annotated on the films; the numbers indicate the molecular weight in kilodaltons (kDa). These markers confirm that the entire area of the SDS-PAGE gel was captured within the frame of the X-ray films.

Supplemental Fig. S2, Matsumoto *el. al.*
