# Supplementary material for: Pyrogallol B-ring enhances catechin binding to the SARS-CoV-2 spike receptor-binding domain to inhibit interaction with ACE2
Source: Sci Rep. 2026 Feb 28;16:11413. doi: 10.1038/s41598-026-41170-6 (PMC13057188; doi:10.1038/s41598-026-41170-6)
Supplement: Supplementary file 3 — Supplementary Information 3. [file 41598_2026_41170_MOESM3_ESM.pdf]

Supplementary Table S1. Primers used for plasmid construction.
All primers used for PCR amplification and vector linearization are listed below. Primers 1 and 2 were used to amplify the *Luc2* gene from pGL4.23. Primers 3 and 4 were used to amplify the *EmGFP* gene from pcDNA6.2/N-EmGFP-DEST. Primers 5 and 6 were used for the linearization of the pT7-IRES His-C vector via PCR. Primers 7 and 8 were used to amplify the *SARS-CoV-2-Spike-C9* gene from pcDNA3.1-SARS2-spike. The 15-bp complementary sequences (homology arms) required for the In-Fusion cloning system are indicated in blue. Detailed cloning strategies are described in the Materials and Methods section.

Primer 1 (Foward primer for Luc2)
AATATGGCCACAACC ATGGAAGATGCCAAAAACATT
Primer 2 (Reverse primer for Luc2)
CTCCATATGGCTAGC TTACACGGCGATCTTGCC
Primer 3 (Foward primer for EmGFP)
AATATGGCCACAACC ATGGTGAGCAAGGGCGAG
Primer 4 (Reverse primer for EmGFP)
CTCCATATGGCTAGC TTACTTGTACAGCTCGTCCATG
Primer 5 (Foward primer for linear pT7-IRES His-C)
GCTAGCCATATGGAGCTCCT
Primer 6 (Reverse primer for linear pT7-IRES His-C)
GGTTGTGGCCATATTATCATC
Primer 7 (Foward primer for SARS-CoV2-Spike-C9)
TTCAGGTGTCGTGAAGGATC CACCATGTTGTCTTCCTGGTCCTGCTG
Primer 8 (Reverse primer for SARS-CoV2-Spike-C9)
CAGGTCGACTCTAGAGGATCC TAAGCGGGAGCGACCTGG
