# Supplementary material for: Pyrogallol B-ring enhances catechin binding to the SARS-CoV-2 spike receptor-binding domain to inhibit interaction with ACE2
Source: Sci Rep. 2026 Feb 28;16:11413. doi: 10.1038/s41598-026-41170-6 (PMC13057188; doi:10.1038/s41598-026-41170-6)
Supplement: Supplementary file 4 — Supplementary Information 4. [file 41598_2026_41170_MOESM4_ESM.pdf]

Supplementary Table S2. Supplementary Table S2. Docking simulations using two control antiviral drugs: simeprevir and remdesivir. Comparison of the ten strongest binding modes among simeprevir, remidesivir, and GCG. The results of the docking analysis performed using the identical protocol as table 1.

Affinity (kcal/mol)			
mode	GCG	simeprevir	remdesivir
1	-7.8	-8.8	-7.2
2	-7.8	-8.3	-7.2
3	-7.8	-8.0	-7.1
4	-7.5	-7.9	-6.9
5	-7.5	-7.9	-6.7
6	-7.5	-7.9	-6.7
7	-7.3	-7.8	-6.6
8	-7.2	-7.8	-6.6
9	-7.0	-7.7	-6.6
10	-7.0	-7.7	-6.5
Average	-7.38	-7.98	-6.81
